# Regulation of Gene Expression by PI3K in Mouse Growth Plate Chondrocytes

**DOI:** 10.1371/journal.pone.0008866

**Published:** 2010-01-25

**Authors:** Veronica Ulici, Claudine G. James, Katie D. Hoenselaar, Frank Beier

**Affiliations:** CIHR Group in Skeletal Development and Remodeling, Department of Physiology and Pharmacology, University of Western Ontario, London, Ontario, Canada; Emory University, United States of America

## Abstract

**Background:**

Endochondral ossification, the process through which long bones are formed, involves chondrocyte proliferation and hypertrophic differentiation in the cartilage growth plate. In a previous publication we showed that pharmacological inhibition of the PI3K signaling pathway results in reduced endochondral bone growth, and in particular, shortening of the hypertrophic zone in a tibia organ culture system. In this current study we aimed to investigate targets of the PI3K signaling pathway in hypertrophic chondrocytes.

**Methodology/Principal Findings:**

Through the intersection of two different microarray analyses methods (classical single gene analysis and GSEA) and two different chondrocyte differentiation systems (primary chondrocytes treated with a pharmacological inhibitor of PI3K and microdissected growth plates), we were able to identify a high number of genes grouped in GSEA functional categories regulated by the PI3K signaling pathway. Genes such as *Phlda2* and *F13a1* were down-regulated upon PI3K inhibition and showed increased expression in the hypertrophic zone compared to the proliferative/resting zone of the growth plate. In contrast, other genes including *Nr4a1* and *Adamts5* were up-regulated upon PI3K inhibition and showed reduced expression in the hypertrophic zone. Regulation of these genes by PI3K signaling was confirmed by quantitative RT-PCR. We focused on F13a1 as an interesting target because of its known role in chondrocyte hypertrophy and osteoarthritis. Mouse E15.5 tibiae cultured with LY294002 (PI3K inhibitor) for 6 days showed decreased expression of factor XIIIa in the hypertrophic zone compared to control cultures.

**Conclusions/Significance:**

Discovering targets of signaling pathways in hypertrophic chondrocytes could lead to targeted therapy in osteoarthritis and a better understanding of the cartilage environment for tissue engineering.

## Introduction

PI3Ks phosphorylate the 3′-OH position of the inositol ring of inositol phospholipids, producing three lipid products: PtdIns(3)P, PtdIns(3,4)P(2) and PtdIns(3,4,5)P(3). These lipids bind to the pleckstrin homology (PH) domains of proteins such as PKB (Akt) and control the activity and subcellular localisation of a diverse array of signal transduction molecules [1]. Akt is a serine-threonine kinase and is one of the main targets positively regulated by PI3K. It transduces signals from numerous extracellular stimuli and controls processes such as glucose metabolism, cell cycle progression, gene expression, protein synthesis and cell survival in a wide variety of cell and tissue systems [2], [3]. While several transcription factors are known to be regulated by Akt, including AP-1, glucocorticoid receptor and E2F [3], our knowledge of the actual genes controlled by this pathway is relatively limited. Some of the reported Akt-regulated genes are GLUT-1, PEPCK, VEGF, Bcl-2 and p27 [3]–[10].

The PI3K/Akt pathway is generally associated with tissue growth. We have shown earlier that inhibition of PI3K signaling results in reduced growth of tibiae [11]. Long bones, such as tibia, grow and elongate through the process of endochondral ossification where skeletal elements are first laid down as cartilage precursors and then this cartilage is replaced by bone [12], [13]. During endochondral bone development, the cartilage template is organized in 4 chondrocyte subpopulations: resting (closest to the articular end of the bone), proliferative (the next zone towards the middle of the bone) (which express type II collagen, Sox family members 5,6,9, etc.), prehypertrophic and hypertrophic (the zones closer to the mineralized area, which is located in the middle of the bone) (expressing collagen X, Mmp13, VEGF etc) [14], [15].

Hypertrophic chondrocytes are localized between proliferating cartilage and bone and form an essential functional interface by facilitating the transition from cartilage to bone and coupling chondrogenesis to osteogenesis and angiogenesis [16]. Hypertrophic chondrocytes express and secrete numerous factors that contribute to this coupling process such as Bone morphogenetic proteins (BMPs), Wnts, and Ihh, all of which are important for osteogenesis, as well as RANKL and VEGF, which promote osteoclast activation and vascular invasion [16], [17].

Hypertrophic differentiation is not only an essential step in endochondral ossification, but it also seems to be a pathological event in early osteoarthritis. For example, it was shown that formation of very early age-related OA-like lesions in the knee is accompanied by expression of chondrocyte differentiation-related genes associated with hypertrophy [18]–[20].

In a previous publication [11] we showed that Akt is activated in the prehypertrophic/hypertrophic zone of the growth plate and that PI3K inhibition reduces hypertrophic differentiation and longitudinal bone growth. In the current study, we aim to identify the target genes of the PI3K/ Akt pathway in differentiated chondrocytes.

## Methods

### Ethics Statement

All animal studies were approved by the Animal Use Subcommittee of the Council of Animal Care at the University at Western Ontario.

### Materials

Chemical reagents were purchased from Sigma, unless stated otherwise. Timed pregnant CD1 mice were purchased from Charles River Laboratories. Cell culture and organ culture medium components and general chemicals were purchased from Sigma and Invitrogen. LY294002 was purchased from Calbiochem. Antibodies were purchased from Abcam (Factor XIIIa- # ab1834 (AC-1A1)), Sigma (anti β-actin clone AC-15) and Santa Cruz (Phlda2 (TSSC3 (E-14) - # sc-66298), HRP conjugated goat anti-mouse - # sc-2005, HRP conjugated donkey anti-goat - # sc-2020) (HRP conjugated goat anti-mouse - # sc-2005). AEC substrate-chromogen was purchased from Dako and cocktail protease and phosphatase solutions for protein harvest from Roche (protease inhibitor cocktail tablets –complete mini- # 836153001), Sigma (phosphatase inhibitor cocktail 2 # P5726-5 ml) and Calbiochem (phosphatase inhibitor cocktail IV # 524628). 10% NuPAGE®Bis-Tris Pre-Cast Gel System from Invitrogen was also used.

### Primary Chondrocyte Culture

E15.5 mouse long bones were dissected, enzymatically digested and the obtained chondrocytes were plated in 6-well NUNC plates at a density of 2.5×10^5^ cells per ml in monolayer and incubated overnight as described [21]. The following day the cells were incubated with fresh medium containing either 10 µM LY294002 or DMSO (equal volume) for an additional 24 hours.

### Organ Culture and Immunohistochemistry

Tibiae were isolated from E15.5 mice and cultured for 6 days in serum-free medium containing either DMSO control or LY294002 inhibitor (10 µM), as described [11]. Medium and treatments were changed every second day. Immunohistochemistry protocols were performed as described [11]. Sections were incubated in 3% H_2_O_2_ for 15 min at room temperature, followed by incubation in preheated (2 min at 100°C) 10 mM sodium citrate solution (pH 6.0) for 30 min at 97°C. They were then blocked with 5% goat serum. Sections were incubated with 0.10 mg/ml factor XIIIa primary antibody over night at 4°C. The UltraVision LP Large Volume Detection System AP Polymer was used to recognize the primary antibody according to manufacturer's instructions. After washing, the HRP (horseradish peroxidase) conjugated polymer complex was visualized by incubation for ∼5 min with AEC (3-amino-9-ethylcarbazole) substrate-chromogen; sections were then counterstained with hematoxylin for 30 seconds, washed and mounted. All images were taken at room temperature with a Retiga EX camera connected to a Leica DMRA2 microscope. Primary image analyses were performed using Openlab 4.0.4 and Photoshop software.

### Protein Extraction

Protein samples were harvested both from primary monolayer chondrocytes and from tibial explants. After 24 hours of primary chondrocyte culture, the medium was replaced with ice-cold PBS; cells were harvested and then centrifuged for 5 min at 4°C and 1000 X *g*. The supernatant was removed and samples were resuspended in ice-cold RIPA lysis buffer containing protease and phosphatase cocktails and stored at −80°C or immediately used for western blotting [22].

Protein samples were also isolated directly from tibial explants after 6 days of incubation with DMSO or 10 µM LY294002. Six bones were combined from each treatment to obtain better protein yield. The explants were washed with PBS and then weighed in order to add a proportional amount of RIPA/cocktail buffer (1 ml of buffer for 3 g of tissue). The tissue samples were then flash-frozen and stored at −80°C; they were homogenized and sonicated before protein quantification with Bicinchoninic Acid (BCA) assay.

### Western Blotting

Western blotting was performed as described [22] with minor modifications. 20–30 µg of proteins were loaded and size-fractioned on a 10% NuPAGE®Bis-Tris Pre-Cast Gel System, followed by gel transfer to a nitrocellulose membrane, using the Invitrogen I-blot system. The membrane was blocked in 5% BSA-TBST buffer for 1 hour and then incubated with primary antibody against factor XIIIa, over-night at 4°C. The membrane was washed in TBST and incubated with 1∶3000 goat anti-mouse IgG –HRP conjugated secondary antibody (Santa Cruz Biotechnology, Santa Cruz, CA). Signals were visualized using the enhanced chemiluminescence Advance Western blot detection system (Amersham Biosciences, Piscataway, NJ) and Alphaimager 2200.

### Microarray Analysis of Primary Chondrocytes

All data is MIAME compliant and the raw data has been deposited in a MIAME compliant database (GEO). Microarray analysis was performed as described [21]–[23]. Total RNA was extracted from cultures treated with DMSO (control) or 10 µM LY294002 for 24 hours, in 3 independent experiments. The variability between trials was minimal (Figure S1). RNA quality and quantity was assessed using the Agilent 2000 Bioanalyzer system and subsequently hybridized to MOE 430 2.0 mouse chips from Affymetrix©, as previously described [21], [22]. Bioanalyses, microarray hybridization, scanning and M.A.S. 5.0 normalization were completed at the London Regional Genomics Facility. Data were deposited in the GEO database (NCBI Accession number: Series GSE8488 for the inhibitor microarray data normalized with GC_RMA algorithm to be used in GSEA analysis; NCBI Accession number: Series GSE15069 for the inhibitor microarray data normalized using the M.A.S 5.0. algorithm for single gene analysis). Following the initial normalization, data was filtered based on reliable signal using the SG1a-1 script (Signal intensity filter) from GeneSpring GX 7.3.1. The default settings were applied: lowest threshold-signal detection intensity  = 50; percentage of conditions in which a signal has to be higher than threshold in order to pass  = 25%, where the experimental condition is represented by samples (replicates) grouped together based on their parameter values (e.g. DMSO treatment condition vs. LY294002 treatment condition). As a result of this analyses, the fold changes reported for different genes in the following results section represent an average of the 3 trials. The microarray data was also normalized using the GC-RMA algorithm in order to perform GSEA analysis [21].

### Microarray Analysis of Microdissected Growth Plate

Microarray analysis was performed as described [21], [22]. Total RNA was extracted from microdissected growth plates from E15.5 mouse tibiae, as described [24], in 3 independent experiments. The growth plates were manually separated into 3 main zones: Proliferative/Resting (Zone I), Hypertrophic (Zone II) and Mineralized (Zone III). RNA extracted separately from each zone was then hybridized to MOE 430 2.0 mouse chips from Affymetrix©, as described above. Data normalization was performed using the GC-RMA algorithm [21]. Data were deposited in the GEO database (NCBI Accession number: Series GSE7685). Data filtering was performed as described above.

### RNA Isolation and Real-Time RT PCR

RNA was isolated from primary chondrocytes in monolayer culture after 24-hour incubation with DMSO or 10 µM LY294002, as previously described [21], [22]. Taqman real-time PCR was performed as described [22], [25], [26] with primers and probe sets from Applied Biosystems. Amplified transcripts were quantified using the standard curve method. Data were normalized to *Gapdh* (Glyceraldehyde 3-phosphate dehydrogenase) mRNA levels and represent averages and SE from direct comparison of LY294002 and DMSO treatments from at least 4 different trials, determined by GraphPad Prism 4 software. The results are presented as fold change between DMSO and LY294002 treatments, using DMSO from each trial as reference. A 2 sample unequal variance t-test was used –with a *p-value <0.05 considered significant. The expression of *Phlda2* was also analyzed by real-time PCR using RNA isolated from the 3 growth plate zones. In this case, One-way analysis of variance with the Newman-Keuls Multiple Comparison Test was used and a *p-value <0.05 was considered significant.

### FatiGO Analysis

Gene lists identified by single gene microarray analysis were compared using the FatiGO web application from BABELOMICS v3.1 ([27], [28], http://www.babelomics.org). FatiGO associates Gene Ontology (GO) terms (functional categories) to a group of genes with respect to a gene set of reference [27]. The Biological Process at level 6 was selected for functional annotations based on the relevance of GO terms at this level for our analyses. Functional categories were organized based on their adjusted p-value, corrected for multiple testing.

### GSEA Analysis

In addition to the single gene analyses, we used Gene Set Enrichment Analysis (GSEA) algorithm, which is a microarray data analysis method that uses predefined gene sets to identify significant biological changes in microarray data sets [21]. GSEA is especially useful when the gene expression changes in a given microarray data set are relatively small [29], [30].

In order to implement the GSEA algorithm, data was normalized by Robust Multichip Analysis using RMAEXPRESS software v.0.4.1 developed by B. Bolstad, University of California, Berkeley as previously described [31]. Logarithmically transformed expression values were used to implement GSEA. The GSEA algorithm was implemented with GSEA v2.0 software [30], [32]. Ranked expression lists were derived from RMAEXPRESS and GeneSpring GX® 7.3.1.

Using an *a priori* defined set of genes (e.g. the C2 and User-defined (UD) gene sets), the aim of GSEA is to determine if the members of these gene sets are randomly distributed throughout the analyzed gene list (e.g. 1.4-fold changes between DMSO and LY294002) or mostly found at the top or bottom of the list. An enrichment score (ES) was calculated for each of these gene sets and it reflects the degree to which a gene set is overrepresented at the extremes (top or bottom) of the entire gene list [30]. ES was normalized for each gene set to account for differences in gene set size, yielding a normalized enrichment score (NES). The false discovery rate (FDR) corresponding to each NES was then calculated [30]. For further analyses we used the gene sets meeting these cut-off requirements: false discovery rate (FDR) <25% and p-value <0.05. Enriched gene sets were identified in both LY294002 and vehicle (DMSO) data. If a high number of functional categories had FDR above 25% cut-off, then the top 20 gene sets were selected for further analysis.

### User Defined (UD) Gene Sets

UD Gene sets were generated by us using the probe set search tool and the molecular function class of Gene Ontology annotations from GeneSpring GX 7.3.1., as described [21]. Probe set redundancy was eliminated in all gene sets using the CollapseDataset function in GSEA. All probe set identifiers were converted to the Human Genome Organization (HUGO) annotations, and probe sets lacking corresponding HUGO annotations were excluded. A total of 90 user-defined gene sets were generated.

### Gene Sets from the Molecular Signature Database

To provide an additional set of functional categories, we used GSEA in combination with C2 gene sets from the GSEA Molecular Signature Database (MgSigDB), as described (James et al, PLoSONE, accepted December 15^th^, 2009). The C2 data base is represented by a collection of gene sets containing information about specific biological processes, metabolic and signaling pathways, chemical and genetic perturbations, disease phenotype and animal models and also gene sets from the biomedical literature. At the time of the analyses, C2 was comprised of 1137 gene sets. The gene sets from the C2 data base are separated in 2 categories: CP canonical pathways- canonical representations of biological processes compiled by domain experts) and CGP (chemical and genetic perturbations- gene set representing genes induced or repressed by the perturbation) (http://www.broad.mit.edu/gsea/msigdb/collections.jsp#C2).

## Results

### Genes Differentially Regulated between DMSO and LY294002 Treatments

We performed microarray analyses to identify genes regulated by the PI3K/Akt pathway in primary chondrocytes. We first filtered the gene list generated with Gene Spring GX 7.3.1 based on fold change between the DMSO and LY294002 treatments. The starting gene list for the fold change filter was represented by probe sets showing a reliable signal. A number of 5035 probe sets was changed at least 1.4-fold between the 2 conditions (DMSO and LY294002). 2703 probe sets were at least 1.4-fold up-regulated under PI3K inhibition with LY294002 while 2332 genes were at least 1.4-fold down-regulated by LY294002. Progressively, 416, 9 and 1 genes were 2-, 5- and 7-fold up-regulated by LY294002, respectively, and 596, 14, 1 genes were 2-, 5-, 7-fold down-regulated by LY294002 (Table 1). Overall the gene expression changes were modest under PI3K inhibition with most of the differentially expressed genes being located in the < 2-fold change category; this finding is not unusual for the PI3K pathway as other authors noticed similar patterns in previous publications [3], [7]. This is one of the reasons we used the 1.4-fold change cut-off, in addition to previous observations from our published microarray data, showing that numerous markers of chondrocyte differentiation and targets of our studied signaling pathways had less than 1.5-fold change in gene expression between experimental conditions [21].

**Table 1:** Number of genes differentially regulated between DMSO and LY294002 treatments.

Fold change	DMSO vs. LY294002 (Total)	DMSO>LY294002	LY294002>DMSO
1.4	5035	2332	2703
2	1012	596	416
5	23	14	9
7	2	1	1
10	0	0	0

### Intersection between Genes Up-Regulated in Zone II and Down-Regulated by LY294002 (List #1)

**Figure 1:**
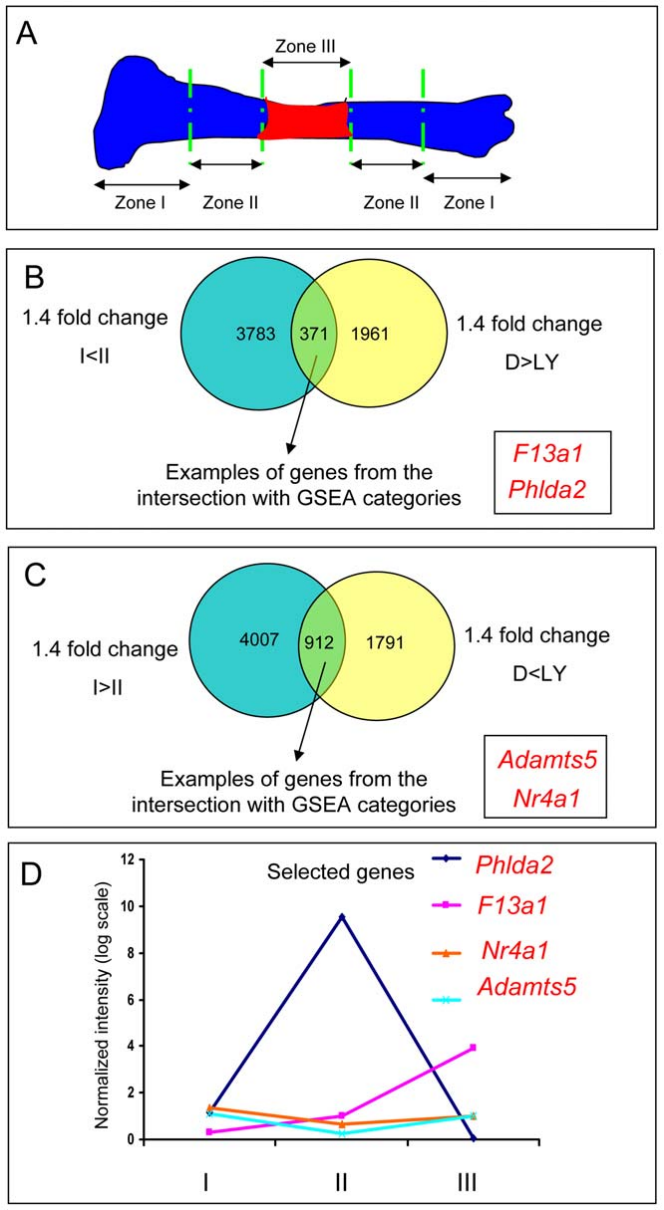
Intersection between genes differentially regulated in DMSO and LY294002 treatments and genes differentially expressed between zone II and zone I. (A) Schematic of the growth plate dissection used for the microdissected growth plate microarray analysis. (B) Intersection between genes up-regulated in zone II and down-regulated in LY294002 Genes 1.4 fold up-regulated in Zone II compared to Zone I (James et al, in revision) were intersected with genes 1.4 fold down-regulated by LY294002. 371 shared genes were identified. (C) Intersection between genes down-regulated in zone II and up-regulated in LY294002.Gene lists up-regulated by LY294002 were intersected with gene lists down-regulated in zone II. 912 probe sets were common to both categories. (D) Expression pattern of selected genes through out the growth plate. *Adamts5* and *Nr4a1* are both decreased in zone II compared to I and III. *Phlda2* is highly increased in zone II compared to I and III. *F13a1* shows increased levels of expression from zone I to zone III.

**Figure 2:**
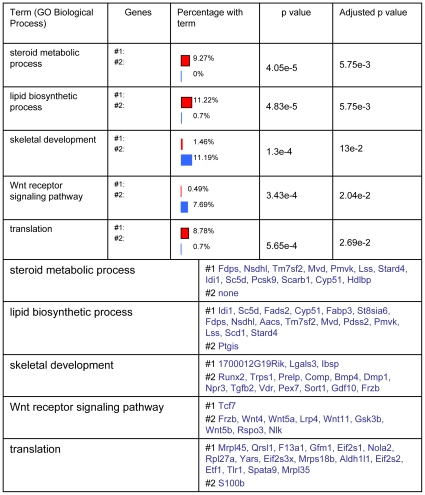
Functional categories identified by GO Biological Process associated with List #1 or with List #2. Functional categories identified from GO biological process at level 6 were presented in the order of the adjusted p-value. The first two categories identified were mostly associated with List #1 (genes both up-regulated in zone II and down-regulated by LY294002) and were represented by “steroid metabolic process” and “lipid biosynthetic process”. The “Translation” category was also highly associated with List #1. In contrast, 11.19% of genes in List #2 (genes both up-regulated in zone II and up-regulated by LY294002) were found in the “skeletal development” class. The percentage with term column represents the percentage of the number of genes in each particular list (List #1 top red: List #2 bottom blue) related to the total number of genes with annotations in both lists.

The main focus of this study was to identify targets of the PI3K pathway in chondrocyte differentiation. Therefore we intersected the genes at least 1.4-fold up-regulated in zone II (hypertrophic zone) compared to zone I (proliferative/resting zone) (James et al, PLoSONE, accepted December 15^th^, 2009.) with the genes at least 1.4-fold down-regulated by LY294002, as potential genes up-regulated by PI3K signaling under physiological conditions. As before, the initial gene set for this experiment was a gene list filtered on the reliable signal. This resulted in the identification of 371 shared genes: both up-regulated during the chondrocyte hypertrophy and down-regulated upon PI3K inhibition (Figure 1B; Figure 2 - #1). Considering that filtering the gene lists on both statistical significance and reliable signal might be too stringent for all the intersections, we decided to use only the reliable signal as the filter for the intersected gene lists.

### Intersection between Genes 1.4-Fold Up-Regulated in Zone II and 1.4-Fold Up-Regulated by LY294002 (List #2)

The probe sets up-regulated in Zone II were also compared to the genes up-regulated by LY294002. 297 probe sets were both 1.4-fold up-regulated in zone II and 1.4-fold up-regulated in the LY294002 treatment (Figure 2 - #2). These genes were both up-regulated in differentiated chondrocytes and up-regulated upon PI3K inhibition with LY294002, opposite to the genes from the previous results section that were down-regulated by LY294002. Therefore, these genes appear to be down-regulated by PI3K during chondrocyte hypertrophy.

### FatiGO Functional Enrichment Identified Metabolic Categories Associated with List #1

We compared the genes from List #1 and List #2 in order to observe which class of genes up-regulated in zone II were down- or up-regulated by LY294002. The previous results have shown that a considerable number of probe sets (297) were up-regulated by LY294002 during chondrocyte differentiation; therefore not all probe sets associated with hypertrophy are also down-regulated by LY294002. We wanted to investigate in more detail which specific biological categories associated with chondrocyte differentiation were also down-regulated by PI3K inhibition.

We observed that a number of metabolic processes from GO Biological Process analysis was associated with List #1. Here we only present the most significant five Biological processes. The first two categories identified by GO biological process at level 6 are represented by “steroid metabolic process” and “lipid biosynthetic process” (Figure 2). 9.27% of the genes in List #1 are represented in the “steroid metabolic process” and 11.22% in the “lipid biosynthetic process”. The “Translation” category was also highly associated with List #1 (8.78% of the genes in List #1).

### Intersection between Genes Down-Regulated in Zone II and Up-Regulated by LY294002

Similar to the previous analysis, gene lists up-regulated by LY294002 were intersected with gene lists down-regulated in zone II, as potential gene targets down-regulated by PI3K activity under normal conditions and with a role in earlier stages of chondrocyte differentiation. 912 probe sets were common to both categories (Figure 1C).

### Gene Set Categories from C2 and UD Databases Enriched in DMSO or LY294002 Conditions

As described in the Methods section, in addition to the single gene analyses we used the GSEA algorithm, which is a microarray data analysis method that uses predefined gene sets and ranks of genes to identify significant biological changes in microarray data sets [21]. After performing the GSEA algorithm in association with the UD and C2 data sets, we found a number of functional categories enriched in either one of the 2 analyzed conditions, DMSO (Table 2, Table S1) and LY294002 (Table S2, S3). The gene sets are organized in tables based on their NES. UD gene sets were found to be associated either with the control condition or the LY294002 treatment. Some examples of the genes sets enriched in the LY294002 phenotype are: **Cartilage**, **TGFB**, **Adipose**, **Wnt3**, **ECM**, while sets enriched in the DMSO phenotype include **Sugar_bind**, **Structure**, **Interleukin-related**, **Hormone**, **Blood** **and Metabolism**. The complete explanation of the C2 categories names can be found in the supplementary tables S1 and S2. Among the C2 gene sets, **Adip_diff_cluster2**, **St_Wnt_Beta_Catenin_Pathway,** **Vegf_Huvec_30min_up** are associated with the LY294002 treatment and **Cholesterol_Biosynthesis**, **IGF_vs_Pdgf_Up**, **Human_Tissue_Placenta** are associated with DMSO.

**Table 2:** User defined (UD) gene sets enriched in DMSO.

NAME	SIZE of gene set	ES	NES	NOM p-val	FDR q-val
SUGAR_BIND	104	0.423	1.858	0.0004	0.014
MUSCLE	198	0.352	1.724	0.0004	0.030
STRUCTURE	151	0.351	1.653	0.0004	0.042
INTERLEUKINRELATED	175	0.343	1.650	<0.0001	0.032
HORMONE	75	0.388	1.626	0.0048	0.032
GLUCONEOGEN	31	0.461	1.581	0.0206	0.040
TNF_RECEPTOR	69	0.368	1.508	0.0134	0.064
**BLOOD**	111	0.326	1.451	0.0117	0.089
CATALYTIC	245	0.274	1.378	0.0059	0.135
PROTEASE_1	269	0.264	1.345	0.0105	0.155
CHEMOKINE	31	0.383	1.335	0.0990	0.151
CYTOKINE	127	0.286	1.308	0.0522	0.166
METABOLISM	196	0.257	1.256	0.0597	0.220
PROTEASE_2	268	0.240	1.219	0.0615	0.261
HEPARIN BIND	37	0.325	1.163	0.2339	0.350
WNT_2	19	0.381	1.159	0.2661	0.336
RGS_RELATED	44	0.312	1.157	0.2326	0.319
NEG_APOPTOSIS	50	0.295	1.119	0.2777	0.382
HEPATOCYTE	19	0.346	1.040	0.3997	0.576
ERK_RELATED	40	0.284	1.039	0.3946	0.548
PHOSPHATASE	473	0.187	1.010	0.4053	0.614
CYTOPLASM	411	0.186	0.991	0.4841	0.644
POS_APOPTOSIS	79	0.234	0.984	0.4839	0.637
ACTIN_CYTOSKEL	38	0.265	0.960	0.5223	0.685
DUSP	20	0.311	0.946	0.5225	0.701
MEMBRANE	260	0.176	0.893	0.7874	0.824
ANGIOGEN	57	0.225	0.885	0.6628	0.813
LIVER_2	260	0.173	0.876	0.8444	0.804
OBL_OCLAST	16	0.246	0.710	0.8549	0.987
APOPTOSIS	39	0.187	0.680	0.9331	0.969

ES, enrichment score.

NES, normalized enrichment score.

NOM p-val, the uncorrected p-value.

FDR q-val, false discovery rate and multiple testing corrections (q-value).

### Intersection of Single Gene Analyses and GSEA

The GSEA functional categories obtained from the previous analysis were then intersected with genes differentially regulated between DMSO and LY294002 treatments from the single gene analysis array data. We intersected the gene sets associated with the DMSO phenotype from the C2 and UD categories with genes 1.4-fold down-regulated in LY294002 treatment (Table S4, S5, Column A). Similarly we intersected gene sets associated with the LY294002 phenotype with genes 1.4-fold up-regulated in the LY294002 treatment (Tables S6, S7, Column A).

### Intersection of Genes in GSEA Categories, 1.4-Fold Down-Regulated by LY294002 Treatment and 1.4-Fold Up-Regulated in Zone II Compared to Zone I

Genes obtained from the intersection of the GSEA C2 or UD categories with genes 1.4-fold down-regulated by LY294002 were further intersected with genes up-regulated in the hypertrophic zone (Zone II) (Table S4, S5, column B). From the final intersection we selected specific genes for confirmation, based on their identification in multiple C2 or UD categories and information from the literature. *F13a1* (coagulation factor XIII, A1 subunit) was identified in both C2 and UD categories: in C2 **Carries_Pulp_High_Up** and **Stossi_Er_Up** and in UD **Blood** and is 2-fold decreased in LY294002 compared to DMSO (Table S4, S5). In addition, factor XIIIa has already been implicated in chondrocyte hypertrophy [33], making it an interesting candidate for further investigation. *Phlda2* (pleckstrin homology-like domain, family A, member 2) was identified in C2 **Nakajima_Mcsmbp_Mast** and **Human_Tissue_Placenta** and is largely decreased in LY294002 (8-fold) compared to DMSO (Table S4, S5). *Phlda2* is also substantially increased in zone II compared to I (∼8-fold). There is no information on the role of Phlda2 in bone growth and our study identified this gene as a novel marker of chondrocyte hypertrophy. Phlda2 is known to be involved in placental growth regulation and contains a PH domain [34], [35].

### Intersection of Genes in GSEA Categories, 1.4-Fold Up-Regulated by LY294002 Treatment and 1.4-Fold Down-Regulated in Zone II Compared to I

Similar to the previous section, genes obtained from the intersection of C2 or UD categories with genes 1.4-fold up-regulated in LY294002 treatment were further intersected with genes down-regulated in zone II of the growth plate compared to zone I (Table S6, S7, column B). *Nr4a1* and *Adamts5* were selected from the resulting gene list for further analyses. *Nr4a1* (nuclear receptor subfamily 4, group A, member 1) was 2.7-fold up-regulated in LY294002 (Table S6, S7) and identified in C2 **AD12_Any_DN**, **AD12_24 hrs_DN** and **Vegf_Huvec_30 min_UP** and UD **2_DNAbind** and **Nucleus_2**. It is also 2-fold decreased in zone II compared to I. Nr4a1 (also known as Nur77) is an orphan member of the nuclear receptor superfamily and exerts opposing biological effects: proliferation, survival and death after induction by extracellular stimuli. The mitogenic activity of Nr4a1 requires DNA binding and translocation to the nucleus [36]. It was suggested that Akt plays a positive a role in the translocation of Nr4a1 from the nucleus to cytoplasm in HEK293T cells [36] and that PI3K inhibition might be related to Nur77 activation leading to apoptosis in HepG2 cells [37]. Our data suggest that there is also regulation at transcriptional level. All these findings make Nr4a1 a potential candidate as a target of the PI3K/Akt pathway in growth plate. *Adamts5* (a disintegrin-like and metallopeptidase (reprolysin type) with thrombospondin type 1 motif, 5 (aggrecanase-2)) was found in UD **Adipose**, **ECM** and **Integrin_rel** and is 4.7-fold down-regulated in zone II compared to I. It is also 1.45-fold up-regulated in response to LY294002 treatment (Table S7). Adamts5 is mostly studied in connection with osteoarthritis and in articular cartilage due to the observation that *Adamts5^−/−^* mice are protected from cartilage degradation in a model of osteoarthritis [38].

### Pattern of Expression of Selected Genes Throughout the Growth Plate

As shown in previous publications from our laboratory, well known chondrocyte differentiation markers, such as *Col10a1*, *Mmp13*, *Ibsp* and *Sox* family members, show the expected expression pattern over the 3 zones in the growth plate microdissection system [24] (James et al, PLoSONE, accepted December 15^th^, 2009.). The expression of the above selected genes was analyzed throughout the growth plate. *Adamts5* and *Nr4a1* are both decreased in zone II compared to I and III (Figure 1C). While *Phlda2* shows substantial increased expression in zone II compared to the other two zones, *F13a1* is also up-regulated in zone III compared to zone II, maintaining high levels of expression in terminal differentiated chondrocytes. Interestingly, *F13a1* shows a similar pattern of expression as genes with an important role in terminal chondrocyte differentiation, such as *Col10a1*, *Ibsp* and *Mmp13* (Figure 1C, James et al, PLoSONE, accepted December 15^th^, 2009

### Confirmation of Array Data by Real-Time PCR

**Figure 3:**
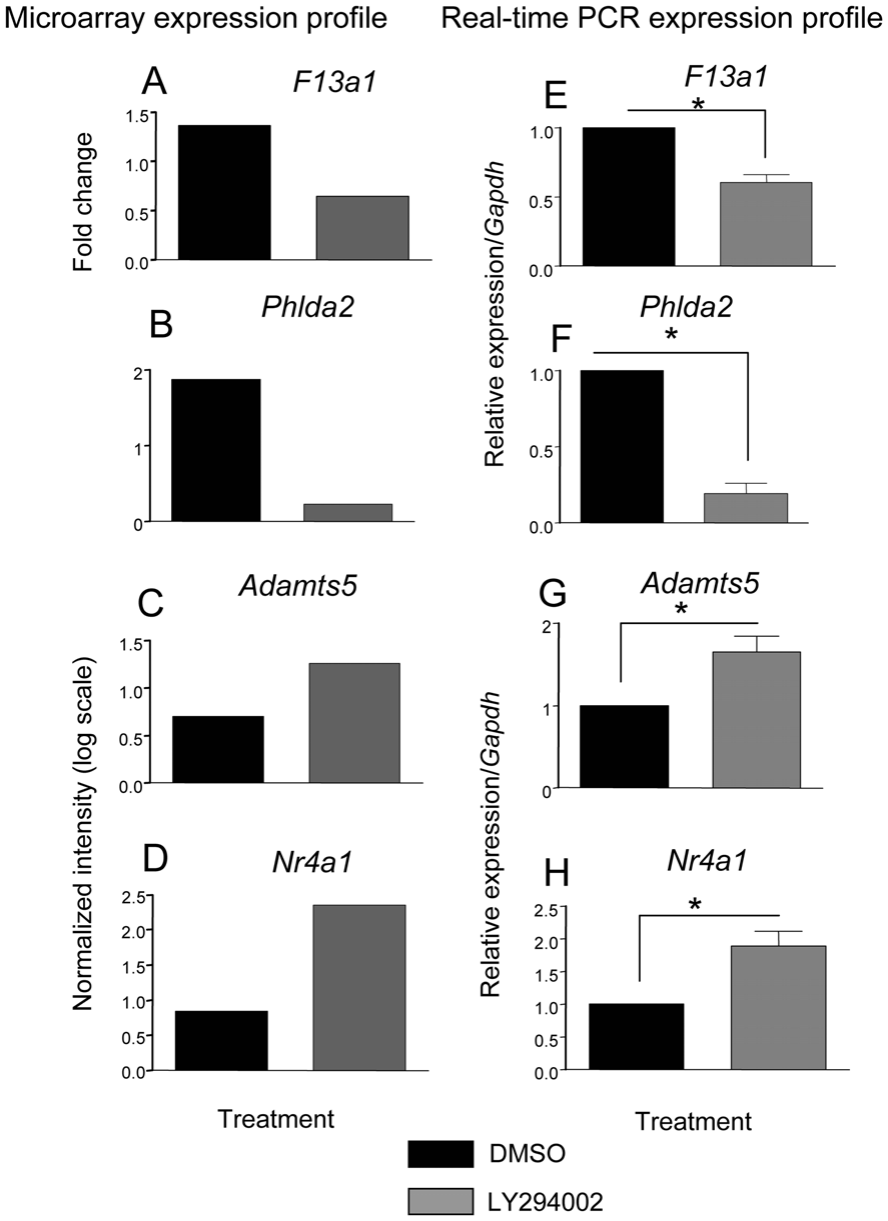
Real-time RT PCR confirmation of selected genes: *F13a1*, *Nr4a1*, *Adamts5*, *Phlda2*. Real-time PCR demonstrates that relative transcript levels for *F13a1* and *Phlda2* were decreased in LY294002 treatment after 24 hours while *Adamts5* and *Nr4a1* transcripts were increased in response to LY294002. Data were normalized to *Gapdh* (Glyceraldehyde 3-phosphate dehydrogenase) mRNA levels. The results represent average fold change between DMSO and LY294002 treatments from 4 independent trials (n = 4). T-test -2 sample unequal variance was used and a *p-value <0.05 was considered significant.

A set of genes was chosen for the real-time validation of the microarray data. Expression of *F13a1* and *Phlda2*, chosen as target genes of PI3K in hypertrophic chondrocyte differentiation, were confirmed by real-time RT PCR. These genes showed a similar trend and fold change compared to the microarray data: *F13a1* (Figure 3E) and *Phlda2* (Figure 3F) were 1.65- and 5-fold down-regulated by LY294002. In contrast, *Nr4a1* (Figure 3H) and *Adamts5* (Figure 3G) were 1.9- and 1.65-fold up-regulated by LY294002, again validating the trends observed in our microarrays.

### Decreased Levels of Factor XIIIa in the Tibial Growth Plate under PI3K Inhibition

**Figure 4:**
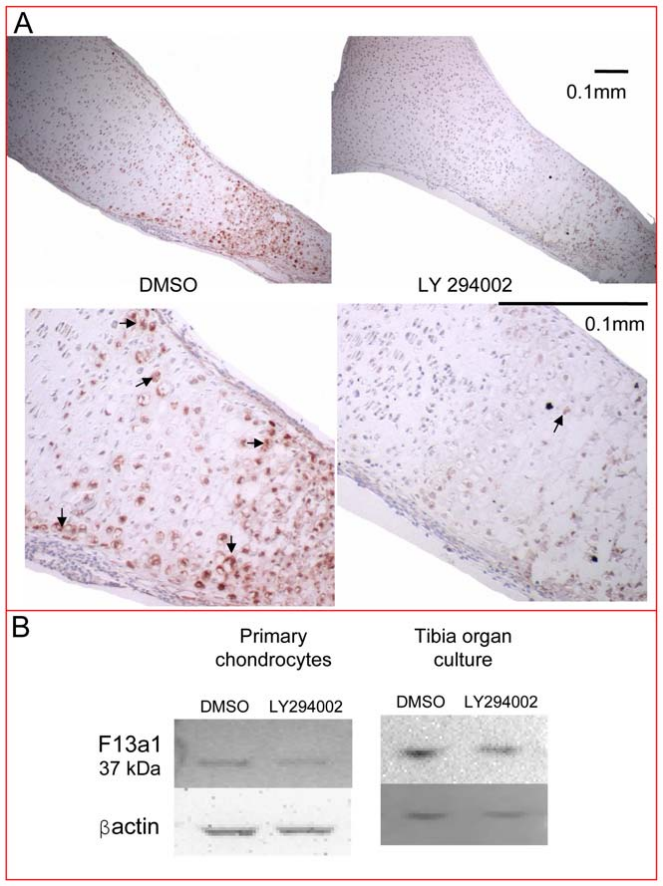
Decreased factor XIIIa protein levels in the tibial growth plate under PI3K inhibition. (A) E15.5 mouse tibiae cultured for 6 days in the presence of DMSO or 10 µM LY294002 were processed for immunohistochemistry. Protein levels of factor XIIIa were decreased in the LY294002-treated growth plates compared to the DMSO control, as shown by the red-brown stain (arrows). (B) Factor XIIIa protein levels were also analyzed by western blotting in both primary cell chondrocyte monolayer cultures after 24 hours and tibiae grown for six days in organ culture, showing decreased factor XIIIa protein levels under PI3K inhibition.

The expression pattern of factor XIIIa has already been analyzed in mammalian and avian growth plates [33], [39], [40] and was shown to be increased in hypertrophic chondrocytes and areas of mineralization. Factor XIIIa levels are also increased in osteoarthritic articular cartilage compared to age-matched normal articular cartilage [39]. For these reasons, we decided to follow up on factor XIIIa as an interesting target of the PI3K pathway in differentiated chondrocytes. To examine regulation of the corresponding protein in the authentic three-dimensional context of the intact growth plate, we cultured E15.5 mouse tibiae for 6 days in the presence of DMSO or 10 µM LY294002 and processed them for immunohistochemistry (Figure 4A). The *F13a1* microarray expression pattern in the growth plate was confirmed at the protein level, with increased levels in the hypertrophic zone (Zone II) compared to the resting/proliferating zone (Zone I) and the maintenance of this expression in the mineralized area (Zone III), adding confidence to the results of the microarray data analysis (Figure 1C). Protein levels of factor XIIIa were markedly decreased in the LY294002-treated tibiae compared to the DMSO control (Figure 4A). Factor XIIIa expression levels were also quantified by western blotting in both primary cell chondrocyte monolayer cultures and tibia organ cultures (Figure 4B), showing that LY294002 treatment results in decreased factor XIIIa protein levels. Interestingly, the factor XIIIa protein size identified in our system was 37 kDa, which was also identified as the cell and tissue specific form of factor XIIIa in chondrocyte and osteoblast cultures, bone tissue and macrophages [41].

### Increased Expression of Phlda2 in Zone II

**Figure 5:**
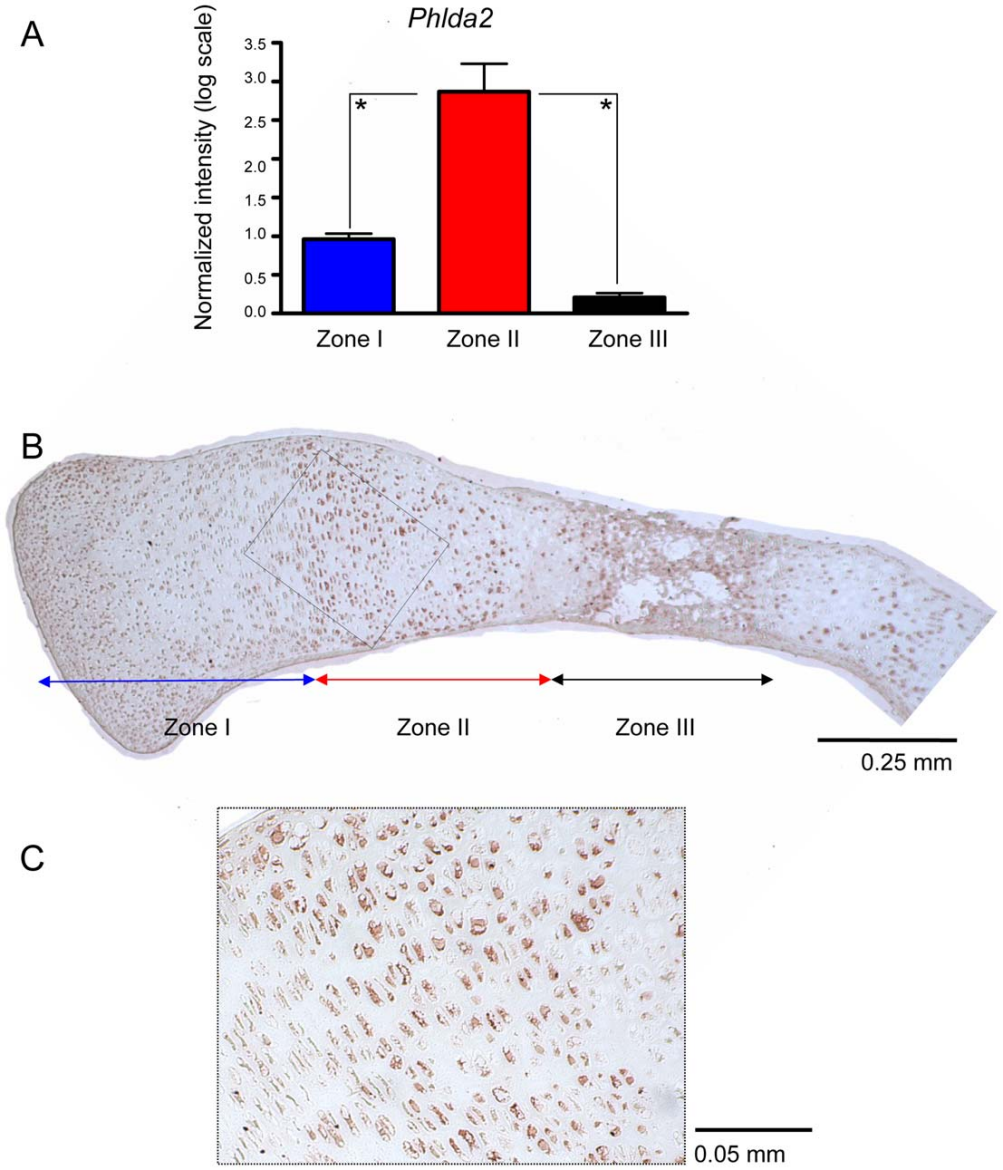
Increased expression of Phlda2 in the hypertrophic zone. (A) RNA was isolated from the 3 growth plate zones. Real-time PCR analyses showed increased expression of *Phlda2* in Zone II compared to Zone I and III. The lowest levels of expression were observed in zone III (13.6 fold change compared to zone II). The presented results are relative to *Gapdh* endogenous control; n = 3, *p<0.05. (B) Expression pattern of Phlda2 within the growth plate after 6 days of culture in the presence of DMSO. Immunohistochemistry analysis showed increased expression of Phlda2 in prehypertrophic/hypertrophic chondrocytes (Zone II) (red-brown stain). (C) The inset shows a higher magnification of Zone II.

The microarray expression pattern of Phlda2 was confirmed by real-time PCR. *Phlda2* was highly expressed in the prehypertrophic/hypertrophic growth plate zone (Zone II) (Figure 5A). The expression pattern in the growth plate was also confirmed by immunohistochemistry (Figure 5B, C).

## Discussion

We have identified that the PI3K/Akt pathway plays a role in hypertrophic chondrocyte differentiation in a previous publication [11]. Here we present a model of target screening in hypertrophic chondrocytes by comparing two different microarray systems, one of primary chondrocytes treated with an inhibitor of the PI3K/Akt pathway and the other one of microdissected growth plate chondrocytes. By intersecting data obtained from these two systems, we identified possible targets of the PI3K/Akt pathway in hypertrophic chondrocyte differentiation.

One of the microarray systems used in this study was represented by the comparison of gene expression in primary chondrocytes isolated from E15.5 mouse long bones treated with the PI3K inhibitor LY294002 or DMSO. Most of the genes differentially regulated between the two treatments showed less than 1.4-fold changes, suggesting that interfering with the PI3K regulation results in small changes in gene expression, as also reported in previous publications [3], [7]. Considering these subtle changes in gene expression, we performed additional microarray data analysis to increase the probability of finding biologically significant targets. For example, subtle changes in the expression of multiple genes involved in the same biological process could still have biologically meaningful consequences, even if changes appear minimal when looking at individual genes. Therefore we implemented GSEA analysis, which was created for data sets showing small changes in gene expression. This method allows for analysis of gene sets grouped in functional categories associated with LY294002 treatment or control phenotypes. These gene sets were then intersected with genes 1.4-fold differentially regulated between DMSO and LY294002 treatments. The resulting genes from this intersection were further intersected with genes differentially expressed between two zones of the microdissected growth plate: zone I and II. The starting gene lists for these intersections were represented by the reliable signal and not the statistical analysis and reliable signal, as used before in other microarray data analyses from our laboratory [21], [22]. This approach was taken due to the multistep process for data analyses in which t-test analyses might be too stringent, eliminating biologically significant data.

We hypothesized that our dual approach of single gene analysis and GSEA algorithm generates highly relevant biological targets of the PI3K/Akt pathway in hypertrophic chondrocytes. The gene set 1.4-fold down-regulated in LY294002 was intersected with gene sets enriched in DMSO phenotype from the UD and C2 data bases. The resulted gene sets were then intersected with genes up-regulated in zone II compared to I (1.4-fold I < II). Using this approach, we aimed to identify genes up-regulated by the PI3K/Akt pathway under physiological conditions in hypertrophic chondrocytes compared to proliferative/resting chondrocytes. The same approach was also used to intersect genes down-regulated by the PI3K/Akt pathway (1.4-fold up-regulated by LY294002), enriched in the LY294002 phenotype in the GSEA analysis and up-regulated in zone I compared to II (1.4-fold I > II). However, the focus of our analysis was on the first intersection, as the major effects of the PI3K/Akt pathway were identified in the hypertrophic zone [11]. Among the genes found in this category, *Phlda2* and *F13a1* were also confirmed by real-time RT-PCR. Factor XIIIa was found mostly intracellular in the prehypertrophic-hypertrophic and mineralized areas, confirming the microdissection microarray data. Factor XIIIa protein levels decreased significantly upon PI3K inhibition, as noticed both in the immunohistochemistry and western-blotting experiments. The expression of factor XIIIa was also decreased in western blots from monolayer and organ cultures, suggesting that our monolayer microarray results are reflective of gene expression changes occurring in the authentic three-dimensional context of the growth plate. The size of the factor XIIIa protein identified in our cultures was 37 kDa, which has been shown to be specific for cartilage/bone tissues and macrophages and is an intracellular form of factor XIIIa [41]. The factor XIIIa was also identified as having a role in osteoarthritis [39], being associated with hypertrophic-like cells. In future experiments it would be of value to test the effects of the PI3K/Akt inhibition on the factor XIIIa in osteoarthritis models. Considering the toxic effects of the LY294002 and the lack of other non-toxic specific PI3K inhibitors for the moment, the system in which this experiment could be performed is an organ culture model of cartilage degradation.

In addition, we identified Phlda2 as a novel marker for hypertrophic chondrocytes, showing high expression in Zone II of the growth plate by both real-time PCR and immunohistochemistry.

We observed that a high number of genes are both up-regulated in zone II compared to zone I (therefore associated with a chondrocyte hypertrophic phenotype) and down-regulated by LY294002 (371 genes). A large number of genes were also up-regulated by LY294002 and in zone II (297 genes); therefore we cannot conclude that the PI3K pathway has a general stimulatory effect on expression of hypertrophic genes. Interestingly, the genes down-regulated by LY294002, in contrast to the ones up-regulated by LY294002, were identified by GO Biological Process analysis as part of a few lipid metabolic groups. It is known that hypertrophic chondrocytes are highly metabolically active cells [42], [43]. In addition, it was suggested before that cholesterol signaling stimulates chondrocyte hypertrophy [23]. Therefore, even if some genes up-regulated in the hypertrophic zone are also up-regulated by LY294002, they do not seem to be associated with the changes in lipid metabolism during hypertrophy.

In this study we propose a model for identification of PI3K/Akt signaling pathway targets in the hypertrophic stage of chondrocyte differentiation. This model could also be expanded to other pathways and organ systems. Due to the combined microarray approach, the possibility of identifying biologically significant targets is high, as demonstrated by our identification of *F13a1* as a target of the PI3K/Akt pathway in hypertrophic chondrocytes and Phlda2 as a novel hypertrophic marker.

## Supporting Information

Figure S1 Heat-maps generated by GSEA analysis. Heat-maps are shown for GSEA C2 and UD functional categories containing genes selected for detailed analysis in the manuscript (F13a1, Phlda2, Nr4a1 and Adamts5). The variability within the DMSO and LY294002 trials was found to be minimal.
(1.55 MB TIF)

Table S1 C2 gene sets enriched in DMSO control
(0.02 MB XLS)

Table S2 C2 gene sets enriched in LY294002 treatment
(0.02 MB XLS)

Table S3 User defined (UD) gene sets enriched in LY294002 treatment
(0.02 MB XLS)

Table S4 Genes found in the intersection of 1.4-fold change I<II and 1.4-fold change D>LY and C2 enriched in DMSO
(0.03 MB XLS)

Table S5 Genes found in the intersection of 1.4-fold change I<II and 1.4-fold change D>LY with UD enriched in DMSO
(0.03 MB XLS)

Table S6 Genes found in the intersection of 1.4-fold change I>II and 1.4-fold change D<LY with C2 enriched in LY294002
(0.03 MB XLS)

Table S7 Genes found in the intersection of 1.4-fold change I>II and 1.4-fold change D<LY and UD enriched in LY294002
(0.04 MB XLS)
