# Supplementary figures and images for: Regulation of Gene Expression by PI3K in Mouse Growth Plate Chondrocytes
Source: PLoS One. 2010 Jan 25;5(1):e8866. doi: 10.1371/journal.pone.0008866 (PMC2810323; doi:10.1371/journal.pone.0008866)

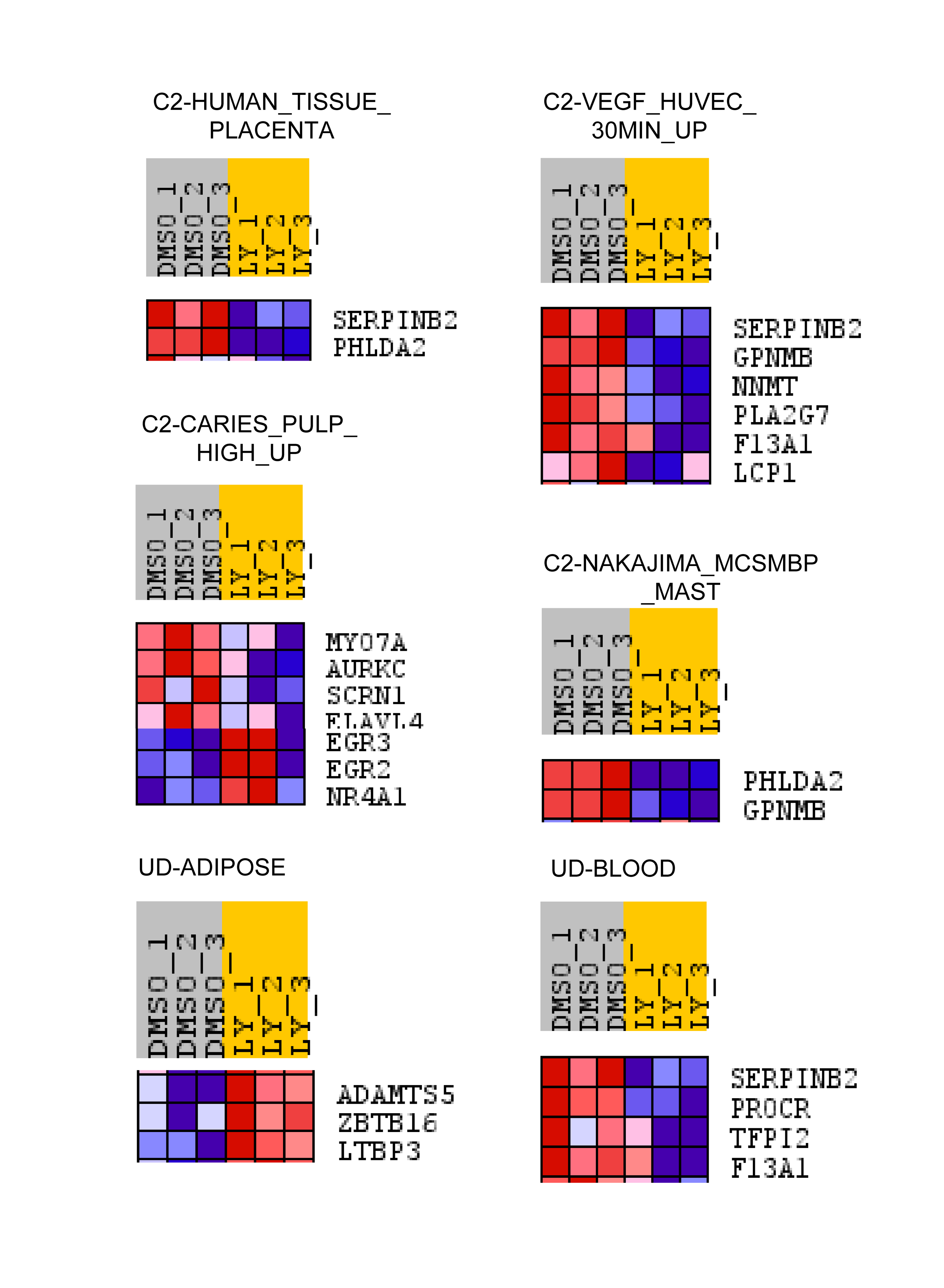

Supplement: Figure S1 — Heat-maps generated by GSEA analysis. Heat-maps are shown for GSEA C2 and UD functional categories containing genes selected for detailed analysis in the manuscript (F13a1, Phlda2, Nr4a1 and Adamts5). The variability within the DMSO and LY294002 trials was found to be minimal. (1.55 MB TIF) [file pone.0008866.s001.tif]
